# Consciousness reflected in the eyes

**DOI:** 10.7554/eLife.35374

**Published:** 2018-03-06

**Authors:** Woon Ju Park, Kimberly B Schauder, Duje Tadin

**Affiliations:** 1 Center for Visual ScienceUniversity of RochesterRochesterUnited States; 2 Department of Brain and Cognitive SciencesUniversity of RochesterRochesterUnited States; 3 Department of Clinical and Social Sciences in PsychologyUniversity of RochesterRochesterUnited States; 4 Departments of Ophthalmology and NeuroscienceUniversity of Rochester School of MedicineRochesterUnited States

**Keywords:** pupil size, pupillometry, consciousness, autism, visual processing, optical illusions, Human

## Abstract

People with higher autistic traits display stronger fluctuations in pupil size when presented with an optical illusion.

**Related research article** Turi M, Burr DC, Binda P. 2018. Pupillometry reveals perceptual differences that are tightly linked to autistic traits in typical adults. *eLife* **7**:e32399. doi: 10.7554/eLife.32399

Most of the tasks that our brain orchestrates in our body are performed outside of our conscious awareness. In addition to breathing, maintaining our balance and keeping our heart beating, these tasks include increasing or decreasing the size of our pupils as our environment becomes darker or lighter. This allows us to see in both darkened rooms and on bright sunny days. Although the area of a pupil can change by as much as a factor of ten (Winn et al., 1994), we are not aware of the small movements in the ocular muscles that open and close our pupils.

Recent studies have painted a complex and fascinating picture of what drives these changes in pupil size. Despite representing the lowest level of visual function, these pupil dynamics can reflect sophisticated processes that are closely linked to our everyday experience (including attention, decision making, and even aesthetic experiences; Binda and Murray, 2015; Hartmann and Fischer, 2014). Therefore, measurements of the pupils, also known as pupillometry, may be used as an indicator for cognitive and perceptual states. This method is also objective and non-invasive, and can, therefore be applied in a wide range of clinical contexts. For example, it enables us to communicate with patients suffering from ‘locked-in’ syndrome – a condition characterized by the paralysis of every muscle except the eye (Stoll et al., 2013).

Now, in eLife, Marco Turi, David Burr and Paola Binda – who are based at research institutes in Pisa, Florence and Sydney – report that fluctuations in pupil size may provide insight into clinical disorders (Turi et al., 2018). The researchers used a well-known optical cylinder illusion that consists of two overlapping sets of black and white dots moving in opposite directions on a 2D plane (Figure 1A). Due to the different speeds of the dots, anyone looking at the dots usually sees a rotating 3D cylinder (Andersen and Bradley, 1998; Video 1 in Turi et al., 2018). However, since the resulting illusion makes it difficult to detect which color is at the front (and, thus, the direction of rotation), our visual system selects one interpretation at any given time. As a result, the perceived rotation switches direction every few seconds (Figure 1B). Such approaches, where the physical stimulus remains constant but our subjective perception changes, have been widely used to study the mechanisms underlying visual awareness (Kim and Blake, 2005).

**Figure 1.**
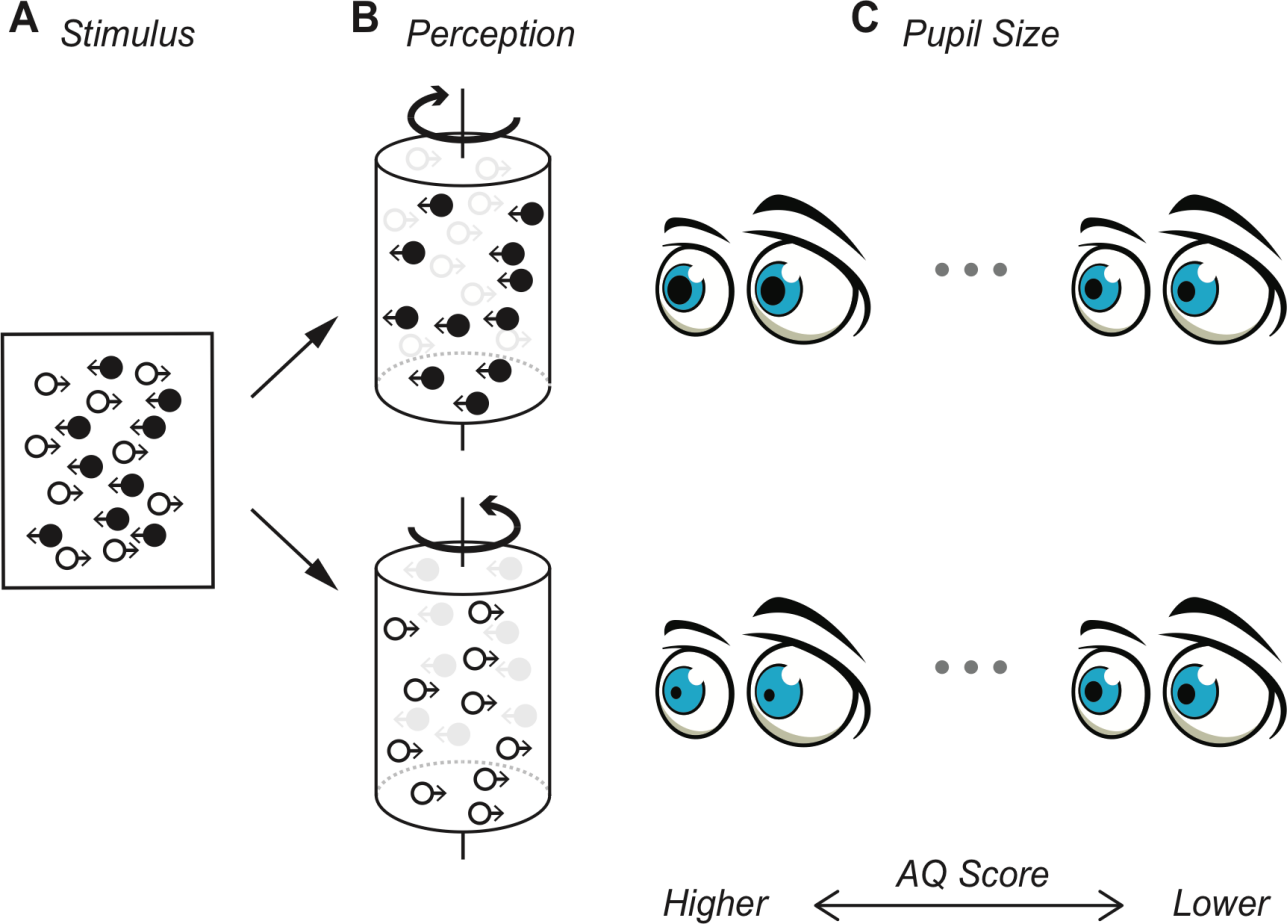
Changes in pupil size depend on subjective visual perception. (**A**) Turi et al. used a visual stimulus in which two sets of dots (white and black) moved in opposite directions (thin arrows). (**B**) To an observer this stimulus appears as a 3D cylinder that rotates in a clockwise direction with the black dots at the front (top), or in a counter-clockwise direction with the white dots at the front (bottom). This perceived direction of rotation switches every few seconds. (**C**) Turi et al. found that pupil size increased when the black dots appeared to be at the front, and decreased when the white dots appeared to be at the front. The size of this change was correlated with Autism-Spectrum Quotient score.

To track any subjective change in perception, the participants reported how they experienced the rotation of the cylinder, while the researchers measured the pupil size. Although the overall intensity of light (the primary factor influencing pupil size) remained constant, the size of the pupils changed: the pupils became larger when the black dots seemed to be in the front, but became smaller, when the white dots appeared to be in front (Figure 1C).

In some participants, the size of the pupils changed substantially between the black and white phases, while in others the changes were only minimal. Turi et al. revealed that these differences strongly correlated with the Autism-Spectrum Quotient (AQ) scores of the participants (Baron-Cohen et al., 2001). The higher the number of autistic traits reported by an individual in the AQ questionnaire, the stronger the changes in pupil size became. This effect accounted for about half of the variance in AQ scores, which is considerably higher than the sizes of the effects reported for other sensory measures (e.g., Ujiie et al., 2015).

Turi et al. argue that the changes in pupil size reflect how individuals process visual information differently. Some people tend to focus on small, defined areas such as the surface at the front, while others concentrate on the entire cylinder. Consequently, people with a more detailed focus should have more fluctuations in pupil size, because their focus alternates between black and white dots. This observation is tightly linked with the long-standing hypothesis that individuals with autism spectrum disorders tend to focus more on the detail rather than the bigger picture (Happé and Frith, 2006).

The findings of Turi et al. provide compelling evidence that the size of a pupil reflects subjective differences in a way that is highly correlated to the reported autistic traits of the participants. The study provides numerous possibilities to investigate higher-level cognitive processes. This can be particularly valuable when studying individuals who may find it difficult to engage in complex behavioral tasks, such as individuals with minimal language skills or deficits in other cognitive abilities. Pupillometry is clearly a powerful tool for characterizing the individual differences in processing visual information and, potentially, for advancing our understanding of autism spectrum disorders.

## References

[bib1] Andersen RA, Bradley DC (1998). Perception of three-dimensional structure from motion. Trends in Cognitive Sciences.

[bib2] Baron-Cohen S, Wheelwright S, Skinner R, Martin J, Clubley E (2001). The autism-spectrum quotient (AQ): evidence from Asperger syndrome/high-functioning autism, males and females, scientists and mathematicians. Journal of Autism and Developmental Disorders.

[bib3] Binda P, Murray SO (2015). Keeping a large-pupilled eye on high-level visual processing. Trends in Cognitive Sciences.

[bib4] Happé F, Frith U (2006). The weak coherence account: detail-focused cognitive style in autism spectrum disorders. Journal of Autism and Developmental Disorders.

[bib5] Hartmann M, Fischer MH (2014). Pupillometry: the eyes shed fresh light on the mind. Current Biology.

[bib6] Kim CY, Blake R (2005). Psychophysical magic: rendering the visible 'invisible'. Trends in Cognitive Sciences.

[bib7] Stoll J, Chatelle C, Carter O, Koch C, Laureys S, Einhäuser W (2013). Pupil responses allow communication in locked-in syndrome patients. Current Biology.

[bib8] Turi M, Burr DC, Binda P (2018). Pupillometry reveals perceptual differences that are tightly linked to autistic traits in typical adults. eLife.

[bib9] Ujiie Y, Asai T, Wakabayashi A (2015). The relationship between level of autistic traits and local bias in the context of the McGurk effect. Frontiers in Psychology.

[bib10] Winn B, Whitaker D, Elliott DB, Phillips NJ (1994). Factors affecting light-adapted pupil size in normal human subjects. Investigative Ophthalmology & Visual Science.

